# Psychiatric side effects of chloroquine in COVID-19 patients: two case reports

**DOI:** 10.11604/pamj.supp.2020.35.24064

**Published:** 2020-06-20

**Authors:** Roukaya Benjelloun, Yassine Otheman, Chafik EL Kettani

**Affiliations:** 1 Faculty of Medicine, Mohammed VI University of Health Sciences (UM6SS), Casablanca, Morocco; 2 Faculty of Medicine and pharmacy, Sidi Mohamed Ben Abdellah University, Fez, Morocco

**Keywords:** Chloroquine, COVID-19, psychiatry, anxiety, hallucination, side effect

## Abstract

Chloroquine and hydroxy chloroquine are widely use in Africa and all over the world as anti-malarial drugs but also in the treatment of chronic inflammatory diseases. Since the outbreak of COVID-19 pandemic, Morocco have included this medication in the COVID-19 treatment guidelines in association with azithromycine. Besides dermatologic problems, ocular impairments and gastro-intestinal effects, quinolines may also cause rarely described psychiatric adverse effects. To our knowledge, there has been no reports of psychiatric side effects of chloroquine or hydroxy chloroquine in the actual context of COVID-19 pandemic. Here, we present the description of two COVID-19 patients who showed psychiatric side effects after chloroquine treatment. One patient expressed psychotic symptoms and the other one experienced acute and intense anxiety. In both cases, and according to Naranjo score, the association between chloroquine and psychiatric side effects was probable.

## Introducton

Antimalarial drugs are widely indicated for different medical conditions and may cause rare but still described psychiatric adverse effects [1]. Behavioral and mood disturbances such as psychosis, anxiety and irritability have been described for decades as possible side effects of quinolines, either they are indicated as curative treatment or as malaria chemo prophylaxy [2,3] There is a renewed interest in antimalarial drugs since they have been used in the treatment of COVID-19 [4], with still ongoing debates on this medication´s efficiency and safety for infected patients [5]. In Morocco, and since the onset of the outbreak, confirmed cases of COVID-19 are systematically put under association of hydroxychloroquine or chloroquine and azithromycine as first line treatment [6]. To the best of our knowledge, psychiatric adverse side effects induced by chloroquine or hydroxhychloroquine in the actual context of COVID-19 outbreak have been until now poorly documented. Here we report two cases of COVID-19 in patients treated with chloroquine associated with azithromycine and who presented with acute onset psychiatric symptoms.

## Patient and observation

**Case 1:** on March 31st, a 46 years old male patient, with a previous history of mild depressive episode treated with vortioxetine since November 2019, and diagnosed with COVID-19 after he presented with dry cough, fever, sore throat and fatigue. The patient had positive result on reverse transcription polymerase chain reaction (PCR) analysis of nose swab specimen. He was admitted into isolated COVID-19 ward and received chloroquine- azithromycine association. He had an electrocardiogram every two days. QT interval was normal to prolonged (corrected QT max: 490ms). On the ninth day of treatment, the patient showed symptoms of distress and insomnia. On the evening of the same day, he showed abrupt onset of psychotic symptoms such as visual hallucinations and incoherent speech, with the outburst of odd behavior and repeated attempts to run away from hospital. The patient´s insight was preserved. The cerebral CT scan was normal. We performed laboratory tests: ionogram, blood count, differential coagulation times, serum glucose, creatinine, sodium, potassium, liver enzymes, HIV, VHB, VHC and Syphilis serologies, who turned out to be normal. We made the decision to interrupt COVID-19 medication protocol as well as vortioxetine, and we initiate amilsulpride at the dose of 100 mg per day. Psychotic symptoms disappeared totally after 48 hours. We tapered off and then stopped amilsulpride within a week. The patient remained asymptomatic, and we evaluated him once in two weeks. On the latest psychiatric assessment (May 20), the patient showed no psychotic symptoms.

**Case 2:** a 35 years old female patient, without any personal or family history of mental illness was diagnosed with non-severe form of COVID-19. The patient was admitted into isolated ward and put under azithomycin/chloroquine association. Three days after the treatment initiation, she expressed symptoms of insomnia, recurrent panic attacks with palpitations, sensation of imminent death, feeling of not being able to control her thoughts, depersonalization and derealization. She also expressed persistent sadness with permanent negative thoughts, and fear of dying, herself and her loved ones. The patient received 2.5 mg of lorazepam spread over 24 hours, with moderate improvement of anxiety symptoms. Five days after the azithomycin-chloroquine initiation, and two days after the onset of anxiety symptoms, the patient interrupted azithtromycin-chloroquine association on her own initiative. Anxiety symptoms disappeared rapidly. We indicated to switch to hydroxychloroquine associated with azithromycin. The patient went under this treatment for six days, and remained asymptomatic. After she left hospital, we followed her up by phone. She did not express any distress, anxiety or other psychiatric symptom.

## Discussion

Moroccan health authorities have set up guidelines for the management of COVID-19 patients including the association of azithromycine and hydroxychloroquine or chloroquine as a first line treatment. According to Moroccan guidelines, chloroquine dosing is 1000mg per day, and hydroxychloroquine dosing is 600mg per day. Both have to be maintained for ten days. Chloroquine and hydroxychloroquine are antimalarial drugs that are also used in the treatment of dermatological, rheumatological and infectious diseases [7]. Various side effects may occur; most of them are gastro-intestinal discomfort, visual disturbance, skin rashes and extrapyramidal symptoms. Rare psychiatric side effects are also described [8]. For both patients, Use of the Naranjo adverse drug reaction scale yielded a score of 7 for chloroquine, which indicates a probable relationship between chloroquine and psychiatric adverse side effects. Regarding Azithromycine, the Naranjo score was 2 for the first patient and 1 for the second patient. Thus, the relationship between psychiatric adversive effects and azithromycine is possible. For the first patient, the Naranjo score for vortioxetine, was 0, which indicates that the relationship with psychiatric side effects is doubtful [9,10].

Most frequent psychiatric side effects expressed by patients under chloroquine or hydroxychloroquine are mania, visual hallucinations, derealization, anxiety, agitation and violent behaviors [11]. Other side effects may also be described. In 1988, Bhatia et al. reported a Capgras syndrome induced by chloroquine in a 9 years old patient [12]. More recently, Collins et al. reported a case of a 15 years old female patient with a catatonic state induced by chloroquine [13]. In the latter case, psychiatric symptoms occurred after hydroxychloroquine usual treatment was replaced by chloroquine. Similarly, our second patient we presented showed intense anxiety under chloroquine and remained asymptomatic after we switched to hydroxychloroquine. Indeed, compared to hydroxychloroquine, chloroquine has a narrower therapeutic index, with higher toxicity and increased risk of side effects [14] The first patient we presented had a previous history of mild depressive episode treated with vortioxetine. Psychiatric history has been identified as a possible risk factor for psychiatric side effects induced by chloroquine/hydroxychloroquine that occur independently of treatment duration and dosing [15]. When patients have undergoing psychiatric condition, antimalarial medication put them at risk of worsening their psychiatric symptoms [16].

Mechanisms of psychiatric side effects of antimalarials remain unclear [17]. In addition to the hypothesis of chloroquine effect on muscarinic and dopaminergic pathways, it was found to be antagonist to both 5-HT3A and 5-HT3B receptors [18]. Since chloroquine belongs to quinolone group, it may also, as well as fluoroqouinolones, act as an angonist to N-methyl-d-aspartate (NMDA) and as an antagonist to gamma-aminobutyric acid (GABA ) receptors [19]. Dopamine, serotonin, acetylcholine glutamate and gamma-aminobutyric acid are all known as neurotransmitters that play a key role in cognition, perception and mood pathways. Thus, chloroquine and hydroxychloroquine can provoke and/or enhance symptoms of anxiety, hallucinations, delusional thoughts or depression [20]. Beyond clinical features of these two case reports, their common thread is the COVID-19 outbreak context. Using hydroxychloroquine or chloroquine as a first line treatment for COVID-19 patients is still controversial. Since these medications are still used in many countries, patients have to be informed of psychiatric potential adverse effects and physicians need to set up preliminary psychiatric screening and careful monitoring in order to detect and manage rapidly potential mood and/or behavior disturbances.

Conclusion Up Down

In Africa, and all over the world, quinolines are widely used in the treatment of various medical conditions. Since the beginning of COVID-19 outbreak, many countries decided to include hydroxy chloroquine and chloroquine to medical treatment guidelines of COVID-19 patients. What is important to keep in mind is: Psychiatric adverse effects due to chloroquine may occur and should be monitored even if they are described as rare. Beside psychotic symptoms, patients may experiment intense anxiety with derealisation and the feeling of imminent death due to chloroquine. Regarding psychiatric adverse effects, hydroxy chloroquine appears to be less at risk than chloroquine. In the specific context of COVID-19 outbreak, potential psychiatric side effects due to chloroquine or hydroxy chloroquine have to been monitored and this dimension has to be included in the global assessment of COVID-19 patients.

## Competing interests

The authors declare no competing interests.

## References

[1] Telgt DS, van der Ven AJ, Schimmer B, Droogleever-Fortuyn HA, Sauerwein RW (2005). Serious psychiatric symptoms after chloroquine treatment following experimental malaria infection. Ann Pharmacother.

[2] Akhtar S, Mukherjee S (1993). Chloroquine Induced Mania. Int J Psychiatry Med.

[3] Tran TM, Browning J, Dell ML (2006). Psychosis with paranoid delusions after a therapeutic dose of mefloquine: a case report. Malar J.

[4] Devaux CA, Rolain JM, Colson P, Raoult D (2020). New insights on the antiviral effects of chloroquine against coronavirus: what to expect for COVID-19. Int J Antimicrob Agents.

[5] Molina JM, Delaugerre C, Le Goff J, Mela-Lima B, Ponscarme D, Goldwirt L (2020). No Evidence of Rapid Antiviral Clearance or Clinical Benefit with the Combination of Hydroxychloroquine and Azithromycin in Patients with Severe COVID-19 Infection. Med Mal Infect.

[6] du Maroc Royaume Répartition des cas par Région. 18-06-2020.

[7] Sahoo S, Kumar M, Sinha VK (2007). Chloroquine-induced recurrent psychosis. Am J Ther.

[8] Garg P, Mody P, Lall KB (1990). Toxic psychosis due to chloroquine--not uncommon in children. Clin Pediatr (Phila).

[9] Naranjo CA, Busto U, Sellers EM (1981). A method for estimating the probability of adverse drug reactions. Clin Pharmacol Ther.

[10] (2012). Adverse Drug Reaction Probability Scale (Naranjo) in Drug Induced Liver Injury. LiverTox: Clinical and Research Information on Drug-Induced Liver Injury.

[11] Bhatia MS, Malik SC (1994). Psychiatric complications of chloroquine. Indian J Psychiatry.

[12] Bhatia MS, Singhal PK, Agrawal P, Malik SC (1988). Capgras syndrome in chloroquine induced psychosis. Indian J Psychiatry.

[13] Collins Gregory B, McAllister Mark S (2008). Chloroquine Psychosis Masquerading as PCP: A Case Report. J Psychoactive Drugs.

[14] Browning DJ (2014). Pharmacology of Chloroquine and Hydroxychloroquine. Hydroxychloroquine and Chloroquine Retinopathy.

[15] Biswas PS, Sen D, Majumdar R (2014). Psychosis following chloroquine ingestion: a 10-year comparative study from a malaria-hyperendemic district of India. Gen Hosp Psychiatry.

[16] Juurlink DN (2020). Safety considerations with chloroquine, hydroxychloroquine and azithromycin in the management of SARS-CoV-2 infection. CMAJ.

[17] Das P, Rai A, Chopra A, Philbrick K (2014). Psychosis likely induced by hydroxychloroquine in a patient with chronic Q fever: a case report and clinically relevant review of pharmacology. Psychosomatics.

[18] Bogaczewicz A, Sobow T (2017). Psychiatric adverse effects of chloroquine. Psychiatria i Psychologia Kliniczna.

[19] Sarro A, Sarro G (2001). Adverse Reactions to Fluoroquinolones. An Overview on Mechanistic Aspects. Curr Med Chem.

[20] Kehr J, Yoshitake T, Ichinose F, Yoshitake S, Kiss B, Gyertyán I (2018). Effects of cariprazine on extracellular levels of glutamate, GABA, dopamine, noradrenaline and serotonin in the medial prefrontal cortex in the rat phencyclidine model of schizophrenia studied by microdialysis and simultaneous recordings of locomotor activity. Psychopharmacology (Berl).

